# Analyzing Modeled Torque Profiles to Understand Scale-Dependent Active Muscle Responses in the Hip Joint

**DOI:** 10.3390/biomimetics7010017

**Published:** 2022-01-20

**Authors:** Fletcher R. Young, Hillel J. Chiel, Matthew C. Tresch, Charles J. Heckman, Alexander J. Hunt, Roger D. Quinn

**Affiliations:** 1 Department of Mechanical and Aerospace Engineering, Case Western Reserve University, Cleveland, OH 44106, USA; rdq@case.edu; 2 Department of Biology, Case Western Reserve University, Cleveland, OH 44106, USA; hjc@case.edu; 3 Department of Biomedical Engineering, Case Western Reserve University, Cleveland, OH 44106, USA; 4 Department of Neuroscience, Case Western Reserve University, Cleveland, OH 44106, USA; 5 Biomedical Engineering, Physical Medicine and Rehabilitation, Physiology, Northwestern University, Chicago, IL 60611, USA; m-tresch@northwestern.edu; 6 Departments of Neuroscience, Physical Medicine and Rehabilitation, Physical Therapy and Human Movement Sciences, Northwestern University, Chicago, IL 60611, USA; c-heckman@northwestern.edu; 7 Department of Mechanical and Materials Engineering, Portland State University, Portland, OR 97201, USA; ajh26@pdx.edu

**Keywords:** biomechanics, locomotion, scaling, torque

## Abstract

Animal locomotion is influenced by a combination of constituent joint torques (e.g., due to limb inertia and passive viscoelasticity), which determine the necessary muscular response to move the limb. Across animal size-scales, the relative contributions of these constituent joint torques affect the muscular response in different ways. We used a multi-muscle biomechanical model to analyze how passive torque components change due to an animal’s size-scale during locomotion. By changing the size-scale of the model, we characterized emergent muscular responses at the hip as a result of the changing constituent torque profile. Specifically, we found that activation phases between extensor and flexor torques to be opposite between small and large sizes for the same kinematic motion. These results suggest general principles of how animal size affects neural control strategies. Our modeled torque profiles show a strong agreement with documented hindlimb torque during locomotion and can provide insights into the neural organization and muscle activation behavior of animals whose motion has not been extensively documented.

## 1. Introduction

Locomotion involves the coordination of many different neuromechanical systems. High-level descending commands and low-level reflex pathways work together to control animal limb segments. The constituent properties of an animal’s biomechanics require its nervous system to be tuned for the tasks that the animal routinely performs [1,2]. Although multi-legged locomotion is a common task performed by animals that vary broadly in size, the fundamental components of the nervous system remain mostly consistent. What changes, then, do nervous system control strategies need to make in order to accommodate size-dependent properties during locomotion?

The magnitude and timing of muscular activity during locomotion is inherently related to the size of an animal. A leg performs different functions during its swing and stance phases of movement and, therefore, requires different muscle activations [3]. During the swing phase, the limb shortens to avoid objects on the ground and moves forward to prepare the foot for touch down [4,5]. Motion is assumed to be dominated by the passive properties of an animal’s limb and is affected by gravity, similar to a pendulum. During the stance phase, in contrast, the leg provides power that propels the animal forward and involves a complex coordination of muscle activity to counteract the destabilizing effects of gravity (similar to an inverted pendulum) and maintain posture [6,7].

The relative contributions of inertial and viscoelastic forces depend on the size-scale of an animal due to the difference in physical properties [8]. For example, during a trot, a horse hindlimb activates muscles briefly in order to initiate the swing phase, and its inertia-dominated limb then swings forward [9,10]. Small animals, such as the rat, compensate for their relatively lightweight limbs by activating muscles throughout the swing phase to move the limb forward against passive viscoelastic forces inherent in their musculature [5]. The relative contributions of inertial forces from the limb and passive viscoelastic forces from the muscles influence the type of control strategies an animal uses during locomotion.

We examined how motor control strategies are altered by the size-scale of an animal, focusing on the continuum from small animals, dominated by viscoelastic forces, to large animals, dominated by inertial forces. In order to simplify our analyses, we used a single rat model to represent multi-muscle dynamics across size-scales rather than constructing multiple models for individual animals (e.g., mouse, cat, horse) at each scale. Limb kinematics for all scaled models followed the same time-varying joint angle profile, representing flat ground walking of the rat. Through the implementation of this model, we analyzed how the relative contributions of the constituent joint torques varied across the animal scales.

This model, however, did not account for differences in limb kinematics used by animals at different scales. The kinematics of distal animal joints in particular vary greatly across scales from the flat foot walking of plantigrade animals to the tiptoe walking found in unguligrade animals [11,12]. The kinematics of the proximal hip joint, however, are relatively consistent across animals and generally consist of a sinusoidal motion. For these reasons, we focused our analysis on the hip joint, which has similar sinusoidal protraction/retraction motion joint excursions in the sagittal plane for animals of different size-scales [13,14,15,16,17,18]. Our results, therefore, are likely to provide general insights into the motor control strategies used by animals at different scales.

In this work, we demonstrate how the size-scale of an animal affects the relative contributions of the constituent joint torques, resulting in the emergence of at least two different modes of control when the size-scale is relatively large or relatively small. These control modes are represented as the active muscle responses required to move the limb into position during swing and stance. Using a multi-muscle neuromechanical model of a rat hindlimb, we predict the size-scale at which animals switch between these control modes. We also show how a single size-scale representation of an animal may not be enough to accurately represent the selected control strategy at each joint.

## 2. Materials and Methods

### 2.1. Limb Dynamics

**Figure 1:**
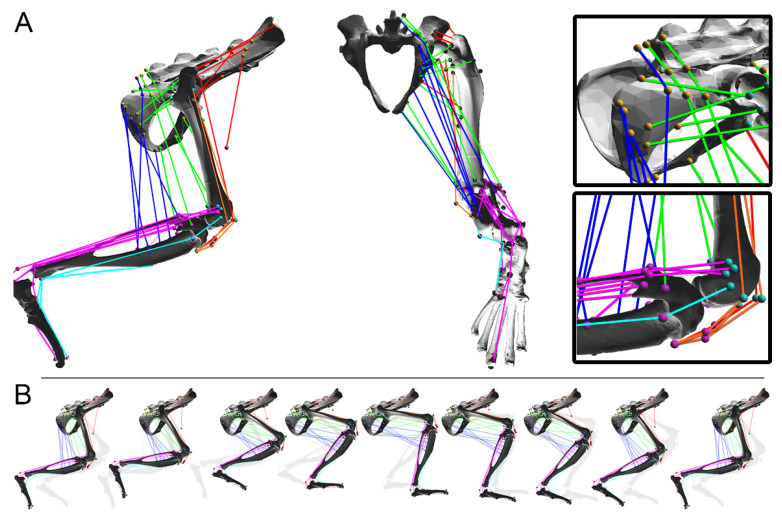
(**A**) A rat hindlimb model with thirty-eight muscles. Joints are modeled as hinges fixed relative to the proximal bone and permitting motion in the sagittal plane. Color is included to visually distinguish muscle groups but has no explicit functional significance. (**B**) The model is moved through a sagittal plane walking pattern that replicates rat locomotion on flat ground. Constituent joint torques, such as passive viscoelastic torques from stretching muscles, are calculated during this walking cycle.

A model of a rat hindlimb was previously developed in the neuromechanical simulation software Animatlab [19,20]. This model, shown in Figure 1A, includes thirty-eight Hill-type muscles, which articulate the pelvis, femur, tibia, and foot. The hip, knee, and ankle are modeled as hinge joints with fixed joint axes relative to the proximal bone. Muscle paths were hand-guided using anatomical data, connecting the bones and using via points to simulate muscle wrapping [21]. The mass and length properties for the hindlimb bones were taken from the literature [22].

Sagittal plane joint motion can be controlled either by activating muscles or by directly actuating the joints with simulated motors. A baseline walking pattern was used for the model at all size-scales, shown in Figure 1B [15]. The focus on sagittal plane walking reduced the computational complexity of the system whilst allowing us to better understand the general relationships between scaling and active muscle responses in a multi-muscle system.

### 2.2. Muscle Dynamics

**Figure 2:**
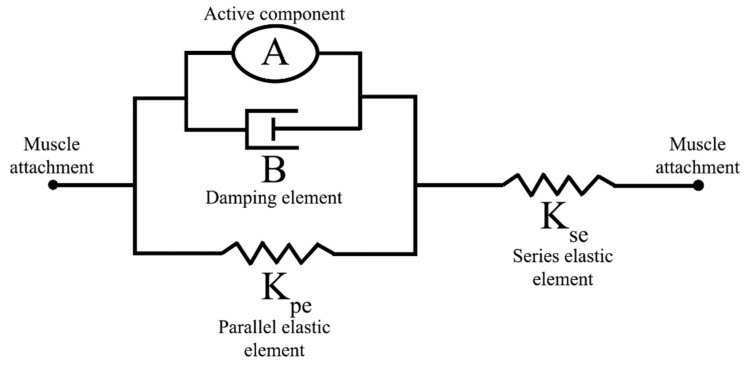
The linear Hill muscle model is implemented in the hindlimb. The spring-damper system replicates the passive and active structures of muscle with three passive viscoelastic components (*K_se_*, *K_pe_*, *B*) and one active component (*A*).

The contraction dynamics of the Hill-type muscle models are described by a spring element in series with a parallel arrangement of a spring, damper, and contractile element, shown in Figure 2 [23]. The passive tension in each muscle is governed by three viscoelastic parameters: the series elastic element (*K_se_*), the parallel elastic element (*K_pe_*), and the damping element (*B*). The contractile element, *A*, contains both length- and stimulus-tension relationships. This results in a tension equation of the form

$$\frac{dT}{dt}=\frac{K_{se}}{B}\left(K_{pe}\left(L-L_{rest}\right)+B\frac{dL}{dt}-\left(1+\frac{K_{pe}}{K_{se}}\right)T+A_{L}A_{M}\right)$$

where *T* represents the muscle tension, *L* is the muscle length, *L_rest_* is the resting muscle length, *A_L_* is the length-tension value, and *A_M_* is the stimulus-tension factor [24]. Length-tension parameters include the resting muscle length and a shaping factor, *L_w_*, for the length-tension curve. The muscle resting lengths were set to coincide with a neutral hanging limb position. The shaping factor was set such that the muscle could generate 70% of the maximum force when at a 50% offset from the resting length [25]. Stimulus-tension parameters were set such that over the activation range of [−60, −40] mV the muscle induced a force output from [0, F_max_]. Maximum muscle forces, F_max_, were taken from the literature [26]. The length-tension and stimulus-tension relationships were represented as

$$A_{L}(L)=1-\frac{{\left(L-L_{rest}\right)}^{2}}{L_{w}^{2}}\;\mathrm{and}\;A_{M}(V)=\frac{ST_{max}}{1+exp\left(S\left(x_{offset}-V\right)\right)}+y_{offset},$$

respectively [20].

The viscoelastic parameters were determined by replicating an experimental hindlimb motion in a simulation. Hindlimb marker data were collected during the intramuscular stimulation of individual muscles in the hanging hindlimb of an anesthetized rat [27]. Analogous muscles in the model were stimulated and the muscle parameters were tuned until the hindlimb joint motion matched the experimental data. Individual muscle parameters were approximated through an adaptive mesh routine to minimize the sum-squared difference between the simulated and experimental joint motion.

### 2.3. Limb Kinetics

**Figure 3:**
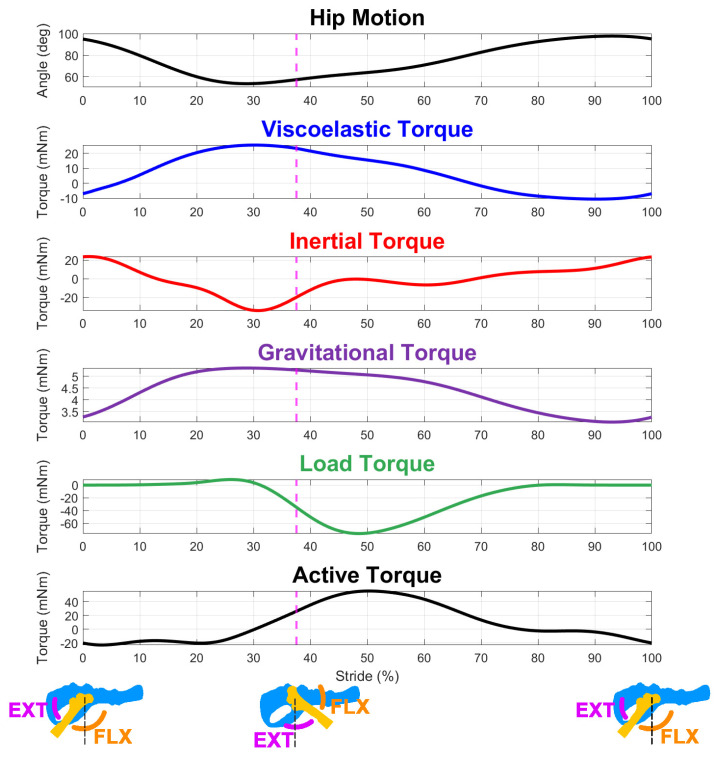
The constituent torques necessary for active muscle coordination are calculated for each joint from the walking model. The dashed line indicates the end of swing and the start of stance; schematics under the data panels show the relative positions of the limb and the extensor/flexor activations associated with its movement. Constituent torques include those required to overcome passive viscoelastic torque due to muscle stretching, inertial torque from moving limb segment masses, gravitational torque from the weight of the limb, and simulated load torque during stance. Active muscle torque counteracts passive hindlimb torques to generate forward motion. The relative contributions of each constituent torque vary depending on the scale of the animals, as indicated by the independent scales of each waveform.

To coordinate walking, animals generate active muscle torque around each joint to counteract passive torques and instigate motion. Constituent torque profiles include those that overcome the inertia of the segment masses in motion, passive muscle forces caused by the stretching of muscles, gravity from the weight of the limb segments, and load torques from ground reaction forces during stance. Constituent passive torque profiles were calculated through inverse dynamics by injecting a kinematic trajectory, calculating the resultant forces generated by the bones, and summing the torques that each muscle produced around the joint. An example of one of these torque profiles is shown in Figure 3 for the model at a rat size-scale. As described in the introduction, we focused on the analysis of torques at the hip.

Inertial torque was calculated by treating the hindlimb as a three-link serial chain of uniform rods connected by hinge joints [28]. Viscoelastic torque was calculated as the spring-damper forces of all muscles crossing a joint multiplied by their dynamic muscle moment arm profiles [29]. Gravitational torque was generated by the hindlimb segments with their weight located at their centers of mass. Load torque, which represents the effect of an animal’s weight, was simulated by applying three-dimensional ground reaction forces to the toe [30]. The joints did not contribute viscoelastic or frictional forces to the model.

### 2.4. Scaling Principles

We implemented a method for scaling the neuromechanical model to quantify the effect that scaling had on active muscle torque trends. Given the length, *L*, the individual model components were scaled to reflect how relative properties were different between animals of a different size. Anatomical positions within the simulation, such as joint coordinates and muscle attachment points, scaled linearly with length, *L*. We assumed that mass scaled with volume, *L*^3^ [31]. The muscle length parameters (*L_r_*, *L_w_*) scaled linearly with *L*. Maximal muscle tension and stimulus-tension parameters scaled with *L*^2^ [32]. We assumed that the spring constants and damping coefficient of the linear Hill muscle model scaled directly with *L* [33,34,35,36].

To accommodate the differences in step frequency across animal sizes, the cycle period was modified such that larger animals walked with a longer cycle time than small animals. The stride cycle period scaled with *L*^0.32^ [37], with the rat-scale model walking at a rate of 1.8 Hz. The relative proportion of the stance and swing phases was maintained across the animal scale.

## 3. Results

### 3.1. Active Torque across Scales

**Figure 4:**
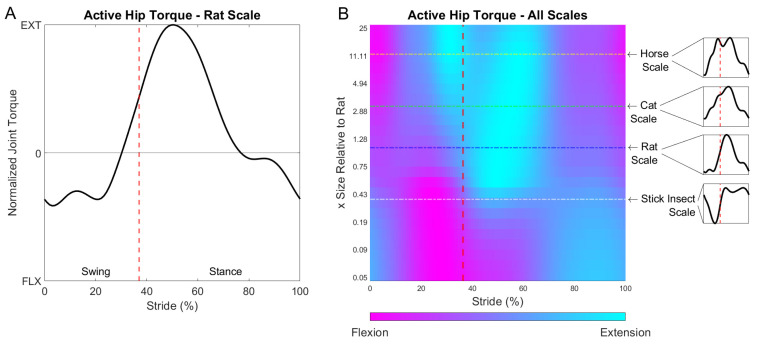
(**A**) Modeled active muscle torque in the hip joint for the model at rat-scale. (**B**) Modeled active muscle torque across a range of size-scales. The flexion-extension profile changes due to size-scale, as indicated by the magenta (flexion) and cyan (extension) coloring. Specific animal sizes are called out to contextualize the general scale of the rat model at which the active hip torques are calculated. The swing-stance transition is represented by the vertical dashed line.

In Figure 4, active muscle torque at the hip is shown during a single stride of locomotion for a range of model scales from 0.05 to 25 times the rat size. Figure 4A shows an active torque stride profile for the rat-scale model. Figure 4B shows the active torque profiles at many size-scales, with individual modeled waveforms in the call-out figures. A number of qualitative changes to the active muscle response can be observed in the figure. For example, the inertia-dominated joint at a horse size-scale exhibited a late swing extension burst that was notably absent in the smaller, viscoelastic-dominated models.

**Figure 5:**
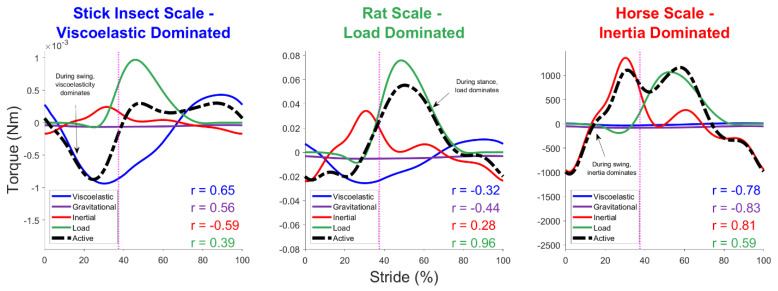
Torque contributions are shown for the modeled hip joint at the scale of a stick insect, rat, and horse. Modeled active muscle torque profiles (black) represent the response of the hindlimb muscles to constituent hindlimb torques. Individual constituent torques are shown for viscoelastic (blue), gravitational (purple), inertial (red), and load (green) forces. As the scale of the model increases, the constituent torque that dominates the active muscle response changes. Linear correlation coefficients, *r*, represent how the constituent torques correlate with active muscle torque.

Constituent joint torques during one stride are shown in Figure 5 for three different model scales that correspond with stick insect, rat, and horse sizes. Active muscle torque had to counteract these constituent torque profiles to create the locomotion trajectory in the model. As the scale of the model increased from a stick insect scale to a horse scale, the active muscle force became less dominated by viscoelastic torque and more dominated by load and inertia. To quantify the dominant values during motion, we used the linear correlation coefficient, *r*, between the total active torque and each separate constituent torque, as shown in each subplot.

Regardless of an animal’s scale, load torque plays a substantial role in the muscular response during stance as the animal supports its body weight and moves forward. At the stick insect scale, strong viscoelastic torques at the hip passively counteracted the load torques, mitigating the need for an active muscular intervention. In contrast, larger animals must dedicate substantial muscle activity to resist a load with little support from their relatively small passive muscle forces.

### 3.2. Dominant Forces during the Swing Phase

**Figure 6:**
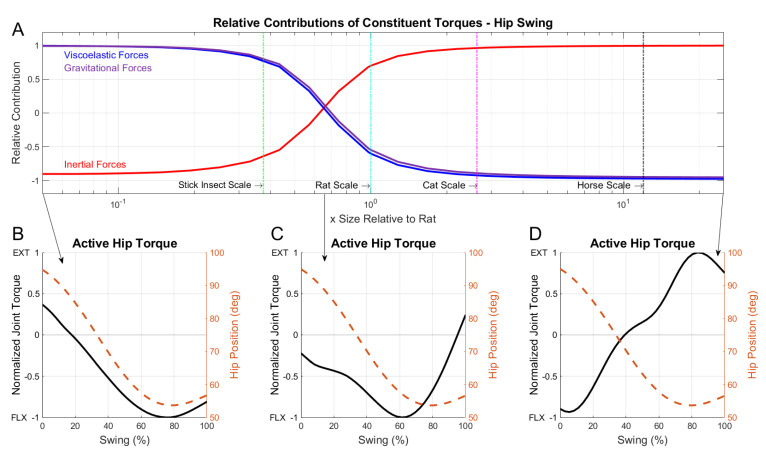
(**A**) Linear correlation coefficient values between constituent torques compared with active torque for the hip joint during swing phase. Viscoelastic (blue), inertial (red), and gravitational (purple) waveforms represent the linear correlation coefficients of each constituent torque relative to active muscle torque. Individual animal sizes are indicated by vertical lines for the model at the scale of a stick insect, rat, cat, and horse. (**B**) Swing phase active joint torques for the hip at three size-scales. Solid lines indicate the normalized joint torque and dashed lines indicated the hip position in degrees. Active joint torque is shown for an animal at 0.05x rat size. The crossover point where viscoelastic and inertial contributions are equal (**C**), and an animal at 25x rat size (**D**). Note that the kinematics of the hip are essentially identical across all three scales but the peak of the active hip torque changes very significantly over the scale. See text.

For a linear increase in the size-scale, there was a nonlinear change in the constituent torques due to the different scaling relationships for each hindlimb component. Depending on the size-scale of the model, the active muscle torque was dominated by either viscoelastic torque or inertial torque. Figure 6A demonstrates the relative contributions of viscoelasticity, gravity, and inertia across a broad range of size-scales that encompassed animals ranging from a stick insect to a horse. At small scales, viscoelasticity and gravity were the driving forces that the active muscle responses counteracted. As the size-scale of the model increased, such as at the horse scale, inertia became the dominating factor.

During the swing phase, the hip joint flexes and the joint angle becomes smaller as the limb moves forward. Depending on the size-scale of the animal, different joint-level behaviors emerged. At small scales, as shown in Figure 6B, the muscles moved in phase with the joint motion by flexing the hip throughout the entirety of the swing. At larger size-scales, the duration of the flexion activity shortened to just an initial burst (Figure 6D). This short flexion burst was followed by a sustained period of extension activity, which slowed the forward inertia of the limb. This “braking” activity was a direct consequence of size-scaling on the neural control. Without this braking behavior, the animal’s heavy limb would continue moving beyond its anticipated touch down position and disrupt locomotion.

### 3.3. Dominant Forces during the Stance Phase

**Figure 7:**
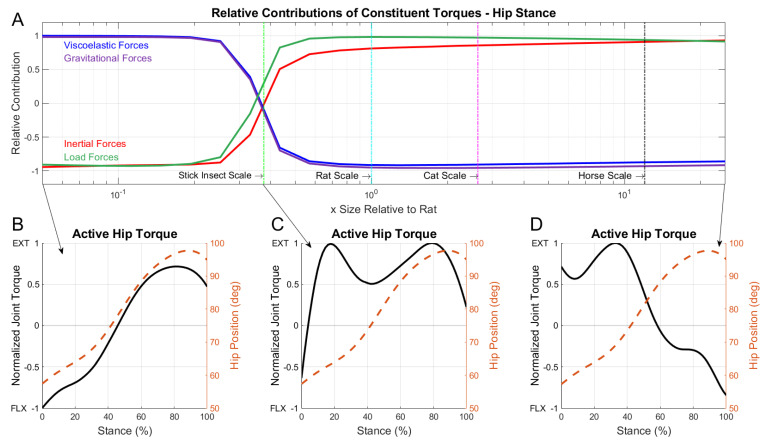
(**A**) Linear correlation coefficient values between constituent torques compared with active torque for the hip joint during stance phase. Viscoelastic (blue), inertial (red), gravitational (purple), and load (green) waveforms represent the linear correlation coefficients of each constituent torque relative to active muscle torque. Individual animal sizes are indicated by vertical lines for the model at the scale of a stick insect, rat, cat, and horse. (**B**) Stance phase active joint torques for the hip at three size-scales. Solid lines indicate the normalized joint torque and dashed lines indicated the hip position in degrees. Active joint torque is shown for an animal at 0.05x rat size. The crossover point where viscoelastic and inertial contributions are equal (**C**), and an animal at 25x rat size (**D**). Once again, the kinematics are almost identical across scales but the relationship between the joint movement and the peak joint torque changes significantly with the scale. See text.

Figure 7A shows the relative force contributions during stance, which had several qualitative differences compared with the swing phase. Most notably, there was a sharper transition between the dominating torques as the size-scale increased. This indicated that the addition of load torque created a more binary relationship for active muscle response where animals were mostly grouped into one of two size-dependent responses.

During stance, the hip joint angle increases as the limb extends to propel the animal forward. At small scales, as shown in Figure 7B, the model suggested that animals extended their hip joint simply by decreasing their active flexion torques and allowing the passive muscle tension to increase the joint angle. Large animals, however, relied on active extension torques to increase the hip joint angle and generate ground reaction forces to sustain the large weight of the animal’s body. Similar to braking during the swing phase, a late stance flexor burst (Figure 7D) slowed the joint’s extension to prepare the limb for toe-off.

### 3.4. Comparison with the Data

**Figure 8:**
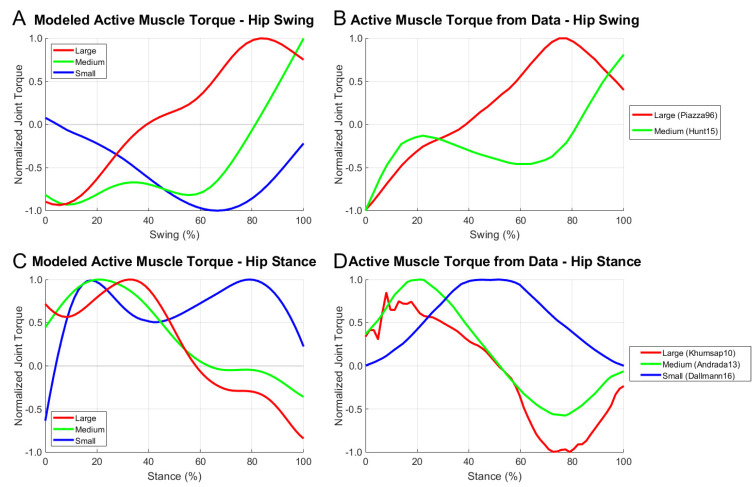
Active joint torque at different size-scales for the modeled rat hip during stance and swing compared with data for animals of different size-scales. Torque values are normalized with respect to the maximal torque during each phase. Modeled animal sizes and coloring represent the general size-scaling of the data subjects above. Animals of each size category were selected based on availability in the literature.

We compared the modeled hindlimb torque of the rat model at different scales with data identified in the literature for a stick insect, rat, and horse [7,38,39]. The modeled torque and data torque, shown in Figure 8, were magnitude- and time-normalized independently to compare the extension-flexion trends across the size-scales. Data were arranged such that extensor and flexor torques were positive and negative, respectively.

Typically, torque data were calculated via inverse kinematics with ground reaction forces because the researchers were interested in the transmission of force throughout the limb during walking. The swing phase torque data were calculated by modeling the inverse dynamics from the motion. The model (Figure 8A) showed a strong agreement with at least one other published model of swing phase torque (Figure 8B). There were little data available for swing phase muscle torque in small animals that could be compared with the model.

Stance phase joint torques showed trend similarities between the model (Figure 8C) and animal data (Figure 8D) as well. At the stick insect scale, there was a similar pattern of high mid-stance muscle activity with minimal activity at swing and stance onsets. This suggested that the phase transitions were largely handled by passive muscle forces rather than active muscular responses. In larger animals, an early stance is characterized by the active extension of the hip followed by a flexion burst for braking.

## 4. Discussion

Using our multi-muscle neuromechanical model, we could predict trends in active torque generation at the hip across size-scales that represented any legged animal. Although we explored a range of 0.05x to 25x the scale of a rat, the model results suggested that there were no notable torque composition transitions outside this range. These predictions allowed us to categorize the animals into three broad groups: those whose locomotion was dominated by inertial forces, those dominated by viscoelastic forces, and those whose locomotion was dominated by a mixture of these constituent forces. By predicting the active muscle torque at a joint, we could make inferences regarding how the nervous system of an animal must change because of the size-scale to accommodate different demands on locomotion.

By comparing our scaled model results with data from animals of similar size-scales, we could better understand how the different biomechanical properties of the model could result in different hip torques as recorded from the animals in the literature. For instance, the stance phase torque data for medium and large animals (green and red traces in Figure 8D) exhibited a strong late stance flexion burst that quickly dissipated as the animal prepared for toe-off. This could indicate that animals reduce their braking during the late stance through a feedforward mechanism to better prepare for toe-off. This same activity was not present in the model, which suggested that it might not predict braking behavior as dramatically as reported in animals. This difference was possibly due to the lack of feedback in the model or through discrepancies in the passive properties of the model, which did not model tendon forces that make large contributions at the extremes of joint motion.

Disparities between the model and animal data could also stem from kinematic differences between the subjects. The small animal responses in Figure 8 demonstrated a clear deviation mid-stance, wherein the data grew to a single large extension value whilst the model exhibited an extension decrease relative to the early and late stance. This was possibly due to the differing morphologies between the rat hindlimb, which has a pendulum-like structure near the body, and the stick insect hindlimb, which extends perpendicular to the body. Even considering these specific active torque differences, it was apparent that the general trend of extension-dominated muscle activation in small animals was present both in the model and the animal data.

**Figure 9:**
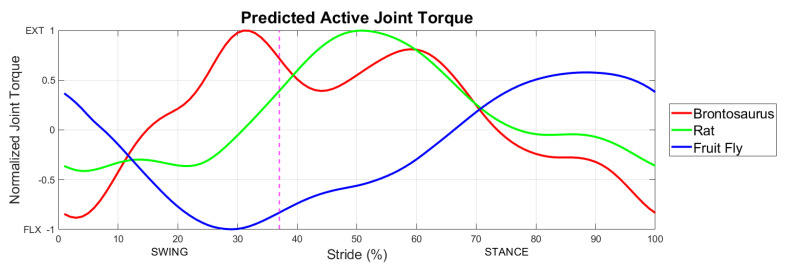
Predicted active joint torque for a rat as large as a brontosaurus [40,41,42] and as small as a fruit fly based on the scaling rules presented.

Current techniques make it difficult to gather torque data from several animals. The fruit fly, for example, is so small that force plate readings are well below modern sensor capabilities. For this reason, the swing phase of Figure 8 lacks sufficiently small animal data for a model comparison. Figure 9 shows modeled active muscle torque for animal scales well beyond those measured in the literature. Active muscle torque during locomotion is indicative of the control strategy employed by the animal’s nervous system based on their size-scale.

Understanding the contrasting control regimes of large and small animals allowed us to predict how joint-level muscle dynamics could change with respect to size-scale. One clear example of the utility of this model was in the emergence of hip braking during swing due to scale. This observation suggested a shift in the neural systems involved in the controlling behavior according to size-scale so that the neural circuitry underlying the locomotion must adapt according to the scale of the animal. These adaptations might be accomplished by alterations in central pattern-generating networks or in afferent feedback pathways [43]. Understanding the scale-dependent responses of signal pathways that regulate behavior, such as hip braking, would enhance our understanding of control and stability due to size-scale and how they are produced by neural systems.

Environmental factors require animals to dynamically select their posture, kinematics, and gait patterns, which can affect the relative contributions of the constituent torques discussed in this work. Inertial torque, notably, increases due to limb speed. It is, therefore, possible that the same animal might shift control regimes from one dominated by viscoelastic torques at slow speeds to another dominated by inertial torques at higher speeds. This multiplicity of the control schemes might be especially relevant for animals at intermediate size-scales such as the rat. For example, a foraging rat might operate in kinetic regimes that use viscoelastic-dominated active muscle torques but would transition to inertia-dominated motion when chased by a predator.

Constituent torque profiles determine whether the active muscle response is in phase or out of phase with the joint motion, a factor which impacts on a joint’s muscular composition [36]. Large animals must prepare to brake their limbs during both stance and swing, which requires a symmetrical muscle composition in both the flexion and extension direction. This symmetric composition may be the result of the straight-leg posture of large animals, in which the limb’s neutral position is near the midrange of the joint. Smaller animals do not require braking and thus require muscles that move the joint to specific positions. The rat’s strong hamstring muscles may have evolved in response to the stance phase requiring a large amount of power to counteract the weight of the animal’s body. An animal’s placement in the size-scale continuum is an important factor in the anatomical makeup of the hindlimb.

Although this work focused only on the impact of size-scale on constituent torque, it would be interesting to consider the effect that activity speed has on the active muscle response. Similarly, different body parts might require distinct control schemes. For instance, human fingers are on a similar scale to rat hindlimbs and so are likely dominated by passive viscoelastic properties whereas arms are likely dominated by inertial properties. These considerations suggest a given animal might require access to different control strategies depending on the actuator being controlled, the environmental conditions, and the task demands.

### Model Limitations

There were several limitations that might reduce the generality of the results and their direct comparison with the experimental animal data. First, this work used the same walking pattern for each size-scale and did not explore the impact of posture on joint torque. Large animals reduce joint torque by using a straighter limb posture, relying on the longitudinal compression strength of bones to support the body [44,45]. Although the general trends between the constituent torques should be the same, the crouched nature of the rat locomotion pattern may suggest higher active muscle torque responses than actually experienced by animals with straight limb postures.

Second, the anatomy of the model was based on a rat for all size-scales rather than developing detailed biomechanical models for different animals at each scale. The relative dimensions of rat anatomy, from bone shapes to muscle insertion points, do not reflect the biomechanics of the different animals. Future work could determine the effects of posture and anatomy along with scale by creating many different biomechanical models and causing them to walk with size-appropriate walking patterns.

Even with these limitations, the predictive capabilities of this work are useful both for biology and robotic design. For biology, this work predicts active muscle torque patterns for animals from which it is currently difficult to gather experimental data. Known neural behavior and pathways in small animals could be used to search for similar pathways in larger animals by looking at how muscle behavior changes during walking. For robotics, this work suggests that torque control strategies for machines must incorporate their size-scale for successful control strategies.
